# Vaccination Challenges and Outcomes in Patients Undergoing Hemodialysis: Experience From a Pharmacist-Led Program in Singapore

**DOI:** 10.7759/cureus.104593

**Published:** 2026-03-03

**Authors:** Pu En Ow Yong, Qian Hui Khor, Sreekanth Koduri, Tharshini Lokanathan, Yee Ding Ng, Debajyoti Roy

**Affiliations:** 1 Pharmacy, Changi General Hospital, Singapore, SGP; 2 Nephrology, Changi General Hospital, Singapore, SGP

**Keywords:** hemodialysis, hepatitis b vaccination, pharmacist-led intervention, pneumococcal vaccination, pneumonia hospitalization, retrospective cohort, seroprotection, singapore

## Abstract

Background

Patients receiving maintenance hemodialysis experience a high burden of infectious morbidity, and vaccination is a key preventive strategy in this population. Despite international guideline recommendations, vaccination uptake and immune response in this population remain suboptimal.

Aims

The study aimed to evaluate hepatitis B virus (HBV) vaccination completion and seroprotection, pneumococcal vaccination coverage, and pneumonia-related hospitalizations among incident hemodialysis patients managed under a pharmacist-led vaccination program.

Design

This retrospective cohort study was conducted between 2019 and 2022 at a tertiary hospital dialysis program in Singapore, where vaccination delivery was integrated into routine hemodialysis care by clinical pharmacists.

Methods

All consecutive incident adult hemodialysis patients managed between January 2019 and December 2022 under a pharmacist-led vaccination model were included. They were evaluated for vaccination uptake, immune response to HBV vaccination, and pneumonia-related hospitalizations within the first year of dialysis initiation. Data were extracted from the hospital’s electronic medical record (EMR) system. As this was a service evaluation of the entire eligible population during the study period, no formal sample size calculation was performed.

Statistical analysis

Categorical variables were analyzed using chi-square testing, with statistical significance defined as a two-sided p-value < 0.05. Results are reported with corresponding chi-square statistics and p-values where applicable.

Results

Among HBV-susceptible patients, just over one-third completed the recommended three-dose vaccination schedule, and approximately half of those achieved seroprotection. Pneumococcal vaccination coverage within the first year of hemodialysis initiation was below 50%. Pneumonia-related hospitalizations were lower among vaccinated patients (2.2%) compared with unvaccinated patients (7.5%) and were statistically significant (p = 0.032).

Conclusions

A pharmacist-led vaccination program was associated with moderate vaccine uptake and reduced pneumonia-related hospitalizations; however, substantial gaps in vaccination completion and immune response persisted. Multicomponent, system-level strategies may be required to enhance vaccine uptake and effectiveness in hemodialysis populations.

## Introduction

End-stage renal disease (ESRD) patients undergoing hemodialysis have a markedly increased risk for infections due to impaired innate and adaptive immunity, chronic inflammation, and repeated vascular access-related exposure to pathogens [1]. The annual incidence of serious infections in hemodialysis patients is estimated to be three to six times higher than in the general population [2]. Hepatitis B virus (HBV) and *Streptococcus pneumoniae* are of particular concern, as they can cause severe morbidity, long-term complications, and mortality in this group [3].

International bodies such as the Centers for Disease Control and Prevention (CDC) [4] and the Kidney Disease: Improving Global Outcomes (KDIGO) [5] recommend early and complete vaccination against HBV, pneumococcal disease, and influenza for all hemodialysis patients. This group of patients is at high risk of contracting hepatitis B due to repeated vascular access and blood exposure. While the overall prevalence of hepatitis B in the USA and Canada is low (0.35% [6] and 0.68% [7], respectively), the prevalence of hepatitis B in immigrants in these countries remains high at 3.9% and 4.2%, respectively. Factors contributing include immigrants from HBV endemic countries and challenges in vaccination uptake, such as fragmented care delivery, absence of designated vaccination coordinators, inconsistent documentation, vaccine hesitancy, and competing medical priorities [8]. This echoes the situation in Singapore, where HBV is endemic and has a prevalence of 3.6% [9]. The pneumococcal vaccination rates in both the US and Singapore for residents above 65 years old were comparable at 64.0% [10] and 22.4% [11], respectively. However, details of pneumococcal rates for hemodialysis patients are not available, although the US resident population between 19 and 64 years old at increased risk for pneumococcal disease was low at 23.0% in 2022 [10], and this is likewise in Singapore as well, given the overall low pneumococcal vaccination rate for the population. This suggests a potential gap in care delivery for this group of patients undergoing hemodialysis who are at higher risk of these infections [12,13].

Pharmacist-led vaccination models have been shown to increase vaccine uptake in chronic disease populations by integrating screening, counselling, and administration into routine care [14-16]. While such models have demonstrated success in North America and Europe, data from Asian settings remain limited. The high prevalence of diabetes-related ESRD and intermediate prevalence of HBV in Singapore highlight the importance of implementing a vaccination model to address the gap. This study aims to evaluate outcomes related to HBV vaccination and pneumococcal vaccination in a pharmacist-led hemodialysis vaccination program.

## Materials and methods

Study design and setting

This retrospective cohort study included all consecutive adult patients initiating maintenance hemodialysis at Changi General Hospital, Singapore, between January 2019 and December 2022. The dialysis program serves a multi-ethnic urban population and delivers in-center hemodialysis. Ethics approval was waived by the SingHealth Centralized Institutional Review Board (CIRB) because this project was classified as a service evaluation and quality improvement initiative, and all data were analyzed in an anonymized manner.

Patient selection

All consecutive incident adult hemodialysis patients, both male and female patients, treated at Changi General Hospital, a tertiary hospital in Singapore, during the study period, were eligible for inclusion. Patients with documented immunity to hepatitis B at baseline, defined as hepatitis B surface antibody (anti-HBs) titers ≥10 IU/L prior to dialysis initiation, and those with documented contraindications to vaccination were excluded from analyses related to HBV vaccination outcomes. Patients without an indication for pneumococcal vaccination based on guideline criteria were excluded from pneumococcal analyses.

Pharmacist-led vaccination intervention

As part of routine hemodialysis care, clinical pharmacists conducted structured reviews of vaccination history during dialysis sessions. Vaccination eligibility was assessed according to recommendations from the CDC [4] and the Singapore Ministry of Health [17]. Eligible patients, defined as those with anti-HBs <10 IU/L prior to dialysis initiation, received HBV vaccination using a high-dose regimen of recombinant hepatitis B vaccine (40 μg Engerix-B® intramuscularly at zero, one, two, and six months). Pneumococcal vaccination consisted of a sequential schedule with pneumococcal conjugate vaccine followed by pneumococcal polysaccharide vaccine, administered according to prevailing guidelines.

Clinical pharmacists coordinated the prescription of vaccines, documentation, and follow-up. Post-vaccination anti-HBs titers were measured four to eight weeks after completion of the HBV primary series. Individualized patient counselling was provided, focusing on infection risk, benefits of vaccination, and the importance of completing multi-dose schedules.

Study outcomes 

Patients were followed for up to 12 months after hemodialysis initiation, and the study focused on the following four outcomes: HBV vaccination completion rate; HBV seroprotection rate; pneumococcal vaccination coverage; and pneumonia-related hospitalizations.

Data collection

The hospital’s electronic medical record (EMR) system, using standardized clinical documentation fields and pharmacy vaccination records, was used to collect data. These collected data included age, sex, primary kidney disease, HBV vaccination completion status, anti-HBs titers following vaccination, pneumococcal vaccination status, and pneumonia-related hospitalizations occurring within the first year of dialysis. Pneumonia-related hospitalizations were identified using the admission diagnoses documented by treating physicians in the EMR.

Statistical analysis

Descriptive statistics were used to summarize patient characteristics and vaccination outcomes. Categorical variables were compared using chi-square tests. Statistical significance was defined as a p-value < 0.05. All analyses were performed using IBM SPSS Statistics for Windows, Version 27 (Released 2019; IBM Corp., Armonk, New York, United States).

Funding 

No external funding was received for this study.

## Results

A total of 381 incident hemodialysis patients were included in the study cohort. Baseline demographic and clinical characteristics of the cohort are summarized in Table 1. The mean age was 62.2 ± 12 years, and 60.6% of patients were male. Diabetic kidney disease was the most common cause of ESRD, accounting for 62.5% of cases (Table 1). 

**Table 1:** Baseline demographics and clinical characteristics of the cohort

Characteristic	Value
Total Patients	381
Age (Mean ± SD, years)	62.2 ± 12
Male (n, %)	231 (60.6%)
Primary Kidney Disease (n, %)	
Diabetic Kidney Disease	238 (62.5%)
Chronic Glomerulonephritis	39 (10.2%)
Hypertensive Nephrosclerosis	25 (6.56%)
Other Causes	79 (20.7%)
Serum albumin level of at least 30g/L	321 (84.2%)

Among the 381 incident hemodialysis patients, 262 patients were eligible for HBV vaccination as their anti-HBs titers were <10 IU/L prior to dialysis initiation and did not have any contraindication to vaccination. A total of 99 patients (37.8%) completed the full three-dose HBV vaccination schedule. Of those who completed the primary series, 50 patients (50.5%) achieved seroprotective anti-HBs titers of ≥10 IU/L (Table 2). 

**Table 2:** Hepatitis B vaccination uptake for patients at risk and their respective immune response (N = 262)

Hepatitis B Vaccination	Study n/N (%)
Received Full 3-Dose Primary Series	99/262 (37.8%)
Received Fewer than 3 Doses	73/262 (27.8%)
Did Not Receive Any Vaccination	90/262 (34.3%)
Seroprotection (anti-HBs ≥10 IU/L) After Primary Series	50/99 (50.5%)

Pneumococcal vaccination within the first year of hemodialysis initiation was documented in 136 of 322 eligible patients, corresponding to a coverage rate of 42.2% (Table 3). Pneumonia-related hospitalizations occurred in seven vaccinated patients (2.2%) compared with 24 unvaccinated patients (7.5%). This difference was statistically significant (chi-square = 4.58, p = 0.032), as shown in Table 3.

**Table 3:** Pneumococcal vaccination uptake for patients at risk and their respective hospitalization outcomes (N = 322)

Pneumococcal Vaccination	Value
Received Vaccine	136/322 (42.2%)
Did Not Receive Vaccine	186/322 (57.8%)
Hospital Admissions for Pneumonia	
- Vaccinated Patients	7/322 (2.2%)
- Unvaccinated Patients	24/322 (7.5%)
Chi-squared value χ²	4.58
Statistical Significance (p-value)	p = 0.032

## Discussion

This retrospective audit demonstrates that, despite the integration of vaccination delivery into routine hemodialysis care through a pharmacist-led model, vaccination uptake and immune response remained suboptimal. HBV vaccination completion and pneumococcal vaccination coverage in this cohort were comparable to rates reported in regional and international studies but remained below targets recommended by international guidelines [4,5,10].

The observed HBV seroprotection rate of approximately 50% among patients completing the primary vaccination series is consistent with another study from the US describing impaired vaccine responsiveness in patients with ESRD (Table 2) [18]. Uremia-associated immune dysfunction, older age, diabetes mellitus, and chronic inflammation are likely contributors to reduced seroconversion in this population. These findings underscore the need for enhanced vaccination strategies, including higher-dose regimens, booster doses, and systematic monitoring of antibody titers.

The pharmacist-led model facilitated opportunistic vaccination during hemodialysis sessions, reduced reliance on external appointments, and improved documentation and follow-up. Nevertheless, persistent barriers were evident. These likely included patient hesitancy, competing clinical priorities during dialysis initiation, and the logistical challenges of completing multi-dose vaccine schedules. Addressing these barriers may require additional system-level interventions beyond pharmacist involvement alone.

Importantly, pneumococcal vaccination was associated with a significantly lower rate of pneumonia-related hospitalizations (Table 3). Although causality cannot be established in this observational design, this finding aligns with existing evidence suggesting that pneumococcal vaccination reduces serious respiratory infections in hemodialysis populations [19]. Reducing infection-related hospitalizations has important implications for patient outcomes and healthcare resource utilization.

Several practical lessons emerge from this experience. Embedding vaccination prompts into electronic dialysis records may reduce missed opportunities. Standing vaccination orders within dialysis protocols could streamline delivery. Prioritizing high-risk patients and using culturally adapted educational materials may further improve acceptance and completion rates.

Limitations

This study has several limitations. The retrospective, single-center design limits generalizability and precludes causal inference. Vaccination status was not randomized, and residual confounding related to age, comorbidity burden, and healthcare utilization may have influenced both vaccination uptake and pneumonia risk. Pneumonia-related hospitalizations were identified from clinical records and may be subject to misclassification. Long-term durability of vaccine-induced immunity was not assessed, and other infection-prevention measures, such as influenza vaccination, were not systematically captured.

## Conclusions

In this real-world audit of incident hemodialysis patients, a pharmacist-led vaccination program was associated with moderate uptake of hepatitis B and pneumococcal vaccines and a lower rate of pneumonia-related hospitalizations among vaccinated individuals. Despite integration into routine dialysis care, vaccination completion and seroprotection rates remained suboptimal, highlighting persistent patient-level and system-level barriers. Multifaceted strategies, including electronic prompts, standing orders, enhanced education, and tailored vaccination schedules, are likely required to optimize protection against vaccine-preventable infections in hemodialysis populations.
